# Sequence Analysis of Feline Coronaviruses and the Circulating Virulent/Avirulent Theory

**DOI:** 10.3201/eid1704.102027

**Published:** 2011-04

**Authors:** Hui-Wen Chang, Herman F. Egberink, Peter J.M. Rottier

**Affiliations:** Author affiliation: Utrecht University, Utrecht, the Netherlands

**Keywords:** Viruses, sequence analysis, feline coronavirus, coronaviruses, FIP, M protein, FIPV, FECV, letter

**To the Editor:** Feline coronaviruses (FCoVs) occur as 2 pathotypes, feline infectious peritonitis virus (FIPV) and feline enteric coronavirus (FECV). FECV is common in cats, causing mild transient enteritis in kittens, but is asymptomatic in adult cats. In contrast, FIPV occurs sporadically but is lethal. It replicates in monocytes and macrophages and rapidly disseminates throughout the body causing systemic immunopathologic disease (*1*–*4*).

The relationship between FECV and FIPV has become a matter of debate. Genetic and animal experimental evidence indicates that FIPV arises by mutation from FECV in the intestinal tract of a persistently infected cat; the virus thereby acquires the monocyte or macrophage tropism that enables it to spread systemically and cause FIP (*5**–**7**,**8*). According to another view, the 2 pathotypes circulate independently in the field. This circulating virulent/avirulent FCoV theory recently was advocated by Brown et al. (*9*). Their conclusion was based on sequence analyses of parts of the viral genome including the matrix (M) gene, phylogenetic analysis of which revealed reciprocal monophyly of the sequences obtained from FIP cases versus those of asymptomatic FECV-infected animals. In addition, the authors suggested 5 aa residues in the M protein to represent potential diagnostic markers for distinguishing virulent FIPV from avirulent FECV (*9*).

To try to verify the findings of Brown et al. (*9*), we determined and analyzed M genes from 43 FCoV genomes, 20 of which came from cats in single-cat households, and 23 from cattery animals. The latter group consisted of 10 asymptomatic healthy cats (FECV; test specimens: feces) and 13 dead cats with FIP confirmed through pathology (FIPV; test specimens: organs, ascites). These animals came from 8 catteries. FECV and FIPV cases were found in 7 (designated A to G); the remaining cattery (H) provided 2 cats with FIP. The genomes from individually living cats were from 15 FIPV- and 5 FECV-infected animals.

Using specific primers (sense 5′-CGTCTCAATCAAGGCATATAATCCCGACGAAG-3′, antisense 5′-CAGTTGACGCGTTGTCCCTGTG-3′), we amplified the same 575-bp M gene fragment as studied by Brown et al. (*9*). GenBank accession numbers for the FCoV M gene sequences determined in this study are HQ738691–HQ738733. When compared by phylogenetic analysis, the nucleotide sequences of FIPV and FECV M genes distributed into paraphyletic patterns rather than in monophyletic clusters (Figure, panel A).

**Figure:**
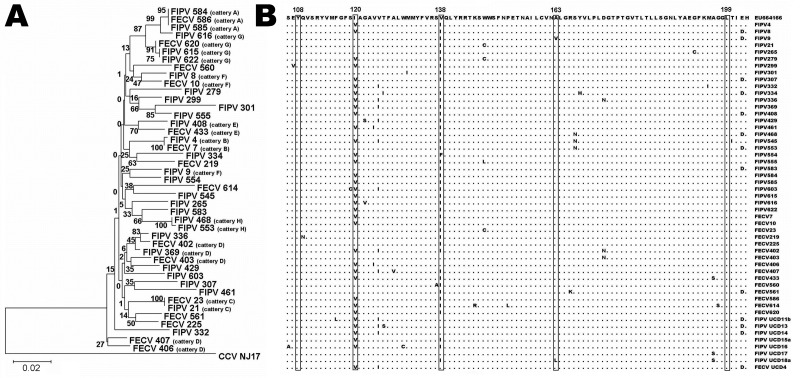
A) Phylogenetic relationships of feline coronaviruses (FCoVs) detected in feces of healthy cats and in organs/ascites of cats with feline infectious peritonitis. Alignment of the matrix (M) gene sequences was used to generate a rooted neighbor-joining tree with the M gene sequence of canine coronavirus strain NJ17 (Genbank accession no. AY704917) as outgroup. Bootstrap confidence values (percentages of 1,000 replicates) are indicated at the relevant branching points. Branch lengths are drawn to scale; scale bar indicates 0.02 nucleotide substitutions per site. Viruses detected in cattery animals are indicated by a cattery designation after the virus identification number. B) Alignment of amino acid sequences of partial M proteins of the FCoVs from panel A, as compared with a feline infectious peritonitis virus (FIPV) reference sequence (top line) published by Brown et al. (*9*) (GenBank accession no. EU664166), and with 8 American FCoV sequences (bottom) published by Pedersen et al. (*8*). The 5 aa residues at positions 108, 120, 138, 163, and 199, suggested by Brown et al. (*9*) as potential diagnostic sites, are boxed.

Thus, as we observed earlier for the 3c gene (*10*), M gene sequences generally clustered according to the cattery from where they originated, irrespective of their pathotype (e.g., FECV 586 and FIPVs 584 and 585 from cattery A; FECV 620 and FIPVs 615 and 622 from cattery G; FECV 10 and FIPV 8 from cattery F). Such a distribution pattern is consistent with the mutation theory, according to which FIPVs originate from FECVs and are thus closely related (*7**,**9*). Exceptions in this picture were FIPV 9 in cattery F and FECVs 406 and 407 in cattery D, presumably caused by multiple FCoV lineages in these open catteries (an open cattery is one in which cats can move in and out, usually for breeding purposes).

We also examined the 5 aa sites in the M protein identified by Brown et al. (*9*) as being potentially diagnostic of FIP. An alignment of the relevant part of the polypeptide sequence, comprising the presumed signature residues at positions 108, 120, 138, 163 and 199, is shown in the Figure, panel B, for all FIPV and FECV genomes sequenced in this study. Within this sample collection, we observed complete sequence conservation at positions 108 and 199, virtually complete conservation (1 difference) at position 163. The 2 aa identities (Val and Ile) found at position 120 and 138 occurred with similar frequencies in FIPV and FECV (position 120: Ile in 16/36 [44%] FIPVs and in 6/14 [43%] FECVs; position 138: Ile in 29/36 [81%] FIPVs and in 12/14 [86%] FECVs). These observations do not indicate the slightest tendency of sequence segregation among the 2 pathotypes. In the alignment of the Figure, panel B, we also included M protein sequences translated from several FCoV genomes from the Americas, 7 FIPV, and 1 FECV (*8*). The comparison does not reveal peculiarities indicative of geographic segregation. Hence, our data do not confirm the diagnostic potential of the M protein sequence nor do they support the suggested role of the membrane protein in FIP pathogenesis (*9*).

Informative as it may be, comparative sequence analysis will eventually not be sufficient to answer the FECV/FIPV question. What will be needed is a reverse genetics system to generate and manipulate the FCoV genome as well as a cell culture system to propagate the viruses, both of which have thus far not been achieved.
